# Supplementary material for: Brown Seaweed Extract (BSE) Application Influences Auxin- and ABA-Related Gene Expression, Root Development, and Sugar Yield in Beta vulgaris L
Source: Plants (Basel). 2023 Feb 13;12(4):843. doi: 10.3390/plants12040843 (PMC9965194; doi:10.3390/plants12040843)
Supplement: Supplementary file 1 [file plants-12-00843-s001.zip › plants-2179777-supplementary.pdf]

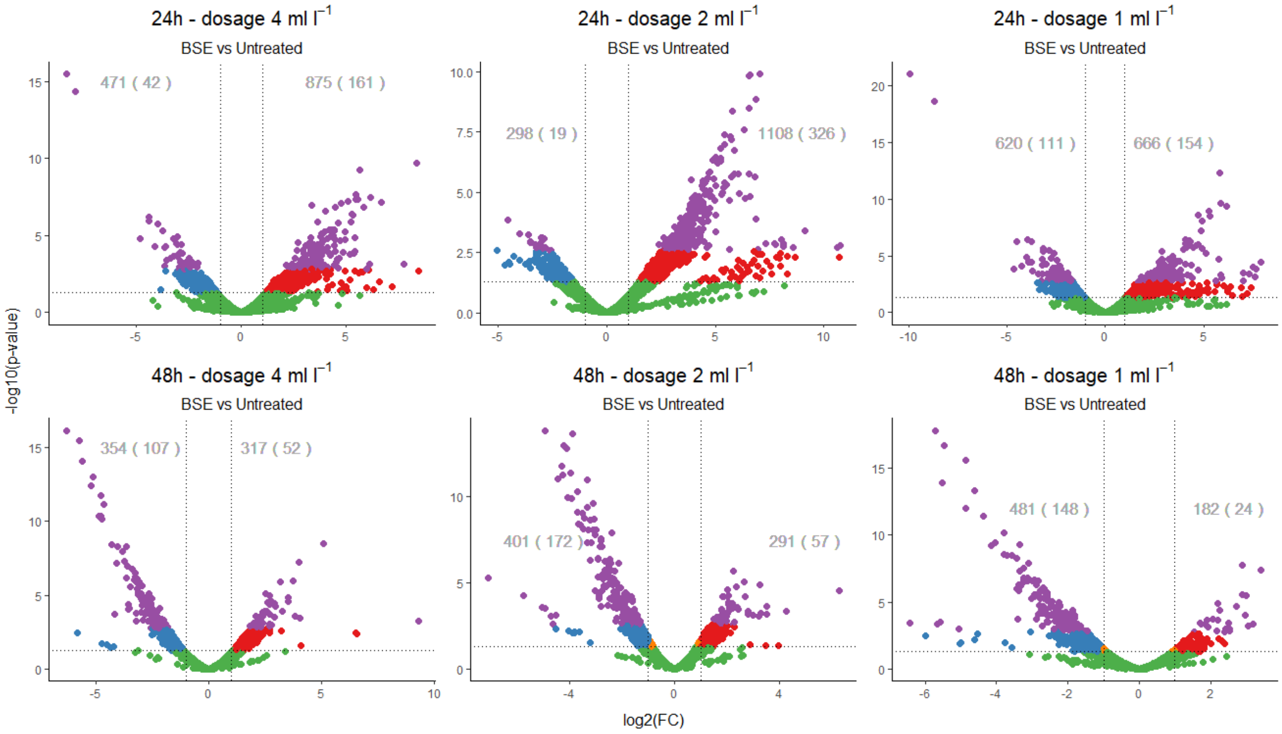

Figure S1. Volcano plots showing DEG genes (p-value<0.05) at 24 h and 48 h after BSE treatment, for each tested BSE concentration. Green and orange dots are genes not DE for the p-value or the FC; Red dots are up-regulated genes (p-value<0.05); Blue dots are down-regulated genes (p-value < 0.05); Purple dots are genes DE according to the adjusted p-value (p-adj<0.1).

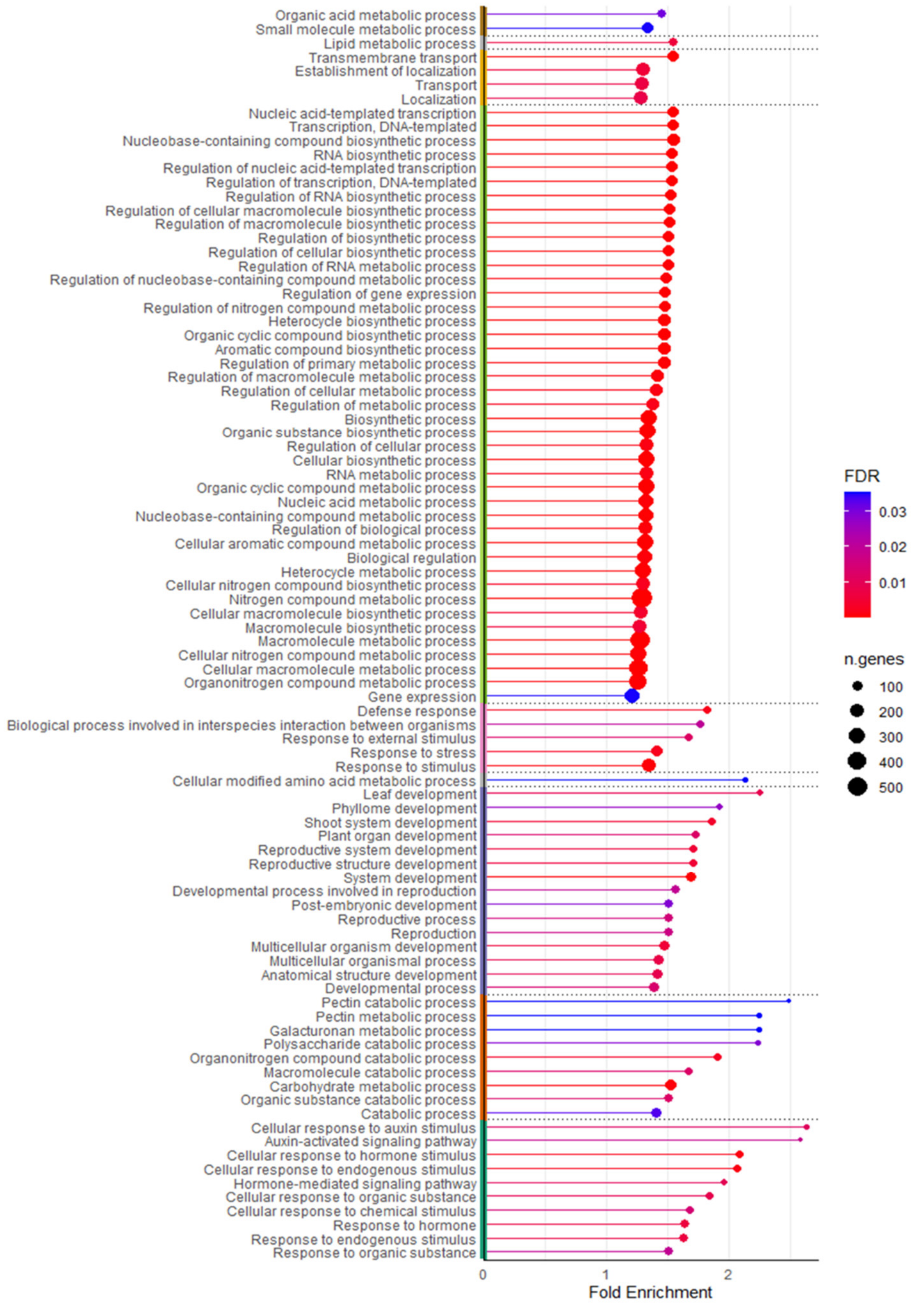

Figure S2. Enrichment chart showing fold enrichment analysis of GO biological processes for up-regulated genes after ANE treatment. The cluster analysis based on similarity is also reported, and a dashed line separates each homogeneous group. In each cluster, pathways are in descending order.

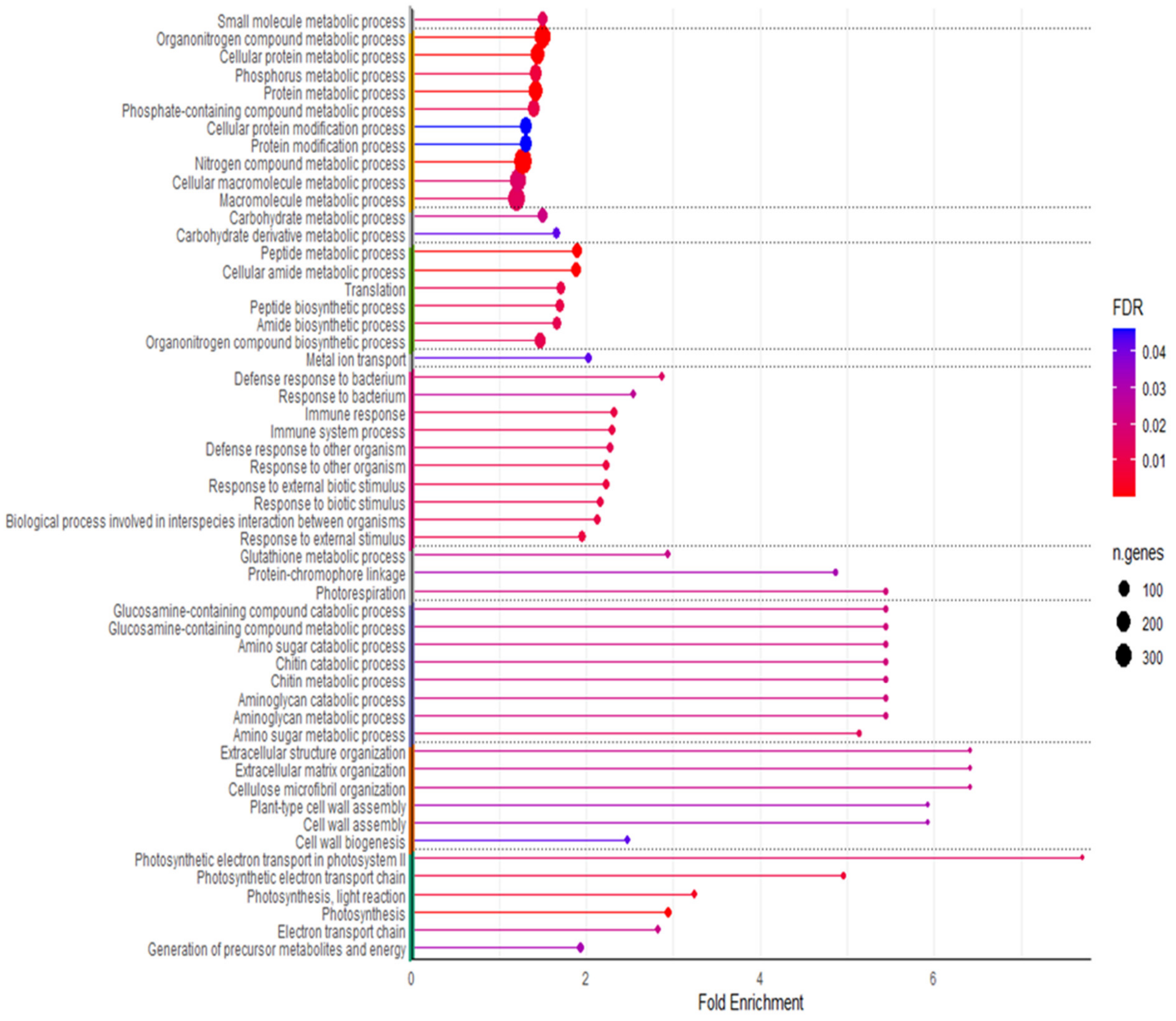

Figure S3. Enrichment chart showing fold enrichment analysis of GO biological processes for down-regulated genes after ANE treatment. The cluster analysis based on similarity is also reported, and each homogeneous group is separated by a dashed line. In each cluster, pathways are in descending order.

One Way Analysis of Variance

Dependent Variable: PYL4

Equal Variance Test (Brown-Forsythe): Passed (P = 0,057)

Group Name	N	Missing	Mean	Std Dev	SEM
0 ml l^{-1} 24 h	9	0	0,766	0,153	0,0511
0 ml l^{-1} 48 h	9	0	0,857	0,239	0,0797
4 ml l^{-1} 24 h	9	0	1,386	0,297	0,0989
4 ml l^{-1} 48 h	9	0	1,312	0,295	0,0982
2 ml l^{-1} 24 h	9	0	0,593	0,114	0,0379
2 ml l^{-1} 48 h	9	0	0,421	0,0752	0,0251
1 ml l^{-1} 24 h	9	0	0,479	0,161	0,0537
1 ml l^{-1} 48 h	9	0	0,730	0,142	0,0474

Source of Variation	DF	SS	MS	F	P
Between Groups	7	8,112	1,159	28,957	<0,001
Residual	64	2,561	0,0400		
Total	71	10,674			

The differences in the mean values among the treatment groups are greater than would be expected by chance; there is a statistically significant difference (P = <0,001).

Power of performed test with alpha = 0,050: 1,000

All Pairwise Multiple Comparison Procedures (Duncan's Method):

Comparisons for factor: **Treatment**

Comparison	Diff of Means	p	q	P	P<0,050
4 ml l^{-1} 24 vs. 2 ml l^{-1} 48	0,964	8	14,463	<0,001	Yes
4 ml l^{-1} 24 vs. 1 ml l^{-1} 24	0,907	7	13,596	<0,001	Yes
4 ml l^{-1} 24 vs. 2 ml l^{-1} 24	0,792	6	11,880	<0,001	Yes
4 ml l^{-1} 24 vs. 1 ml l^{-1} 48	0,656	5	9,831	<0,001	Yes
4 ml l^{-1} 24 vs. 0 ml l^{-1} 24	0,620	4	9,297	<0,001	Yes
4 ml l^{-1} 24 vs. 0 ml l^{-1} 48	0,529	3	7,931	<0,001	Yes
4 ml l^{-1} 24 vs. 4 ml l^{-1} 48	0,0733	2	1,100	0,440	No
4 ml l^{-1} 48 vs. 2 ml l^{-1} 48	0,891	7	13,363	<0,001	Yes
4 ml l^{-1} 48 vs. 1 ml l^{-1} 24	0,833	6	12,497	<0,001	Yes
4 ml l^{-1} 48 vs. 2 ml l^{-1} 24	0,719	5	10,780	<0,001	Yes
4 ml l^{-1} 48 vs. 1 ml l^{-1} 48	0,582	4	8,731	<0,001	Yes
4 ml l^{-1} 48 vs. 0 ml l^{-1} 24	0,547	3	8,198	<0,001	Yes

4 ml l^{-1} 48 vs. 0 ml l^{-1} 48	0,456	2	6,831	<0,001	Yes
0 ml l^{-1} 48 vs. 2 ml l^{-1} 48	0,436	6	6,532	<0,001	Yes
0 ml l^{-1} 48 vs. 1 ml l^{-1} 24	0,378	5	5,665	<0,001	Yes
0 ml l^{-1} 48 vs. 2 ml l^{-1} 24	0,263	4	3,949	0,011	Yes
0 ml l^{-1} 48 vs. 1 ml l^{-1} 48	0,127	3	1,899	0,211	No
0 ml l^{-1} 48 vs. 0 ml l^{-1} 24	0,0911	2	1,366	0,338	Do Not Test
0 ml l^{-1} 24 vs. 2 ml l^{-1} 48	0,344	5	5,165	0,001	Yes
0 ml l^{-1} 24 vs. 1 ml l^{-1} 24	0,287	4	4,299	0,006	Yes
0 ml l^{-1} 24 vs. 2 ml l^{-1} 24	0,172	3	2,583	0,089	No
0 ml l^{-1} 24 vs. 1 ml l^{-1} 48	0,0356	2	0,533	0,708	Do Not Test
1 ml l^{-1} 48 vs. 2 ml l^{-1} 48	0,309	4	4,632	0,003	Yes
1 ml l^{-1} 48 vs. 1 ml l^{-1} 24	0,251	3	3,766	0,013	Yes
1 ml l^{-1} 48 vs. 2 ml l^{-1} 24	0,137	2	2,049	0,152	Do Not Test
2 ml l^{-1} 24 vs. 2 ml l^{-1} 48	0,172	3	2,583	0,089	No
2 ml l^{-1} 24 vs. 1 ml l^{-1} 24	0,114	2	1,716	0,229	Do Not Test
1 ml l^{-1} 24 vs. 2 ml l^{-1} 48	0,0578	2	0,866	0,542	Do Not Test

A result of "Do Not Test" occurs for a comparison when no significant difference is found between two means that enclose that comparison. For example, if you had four means sorted in order, and found no difference between means 4 vs. 2, then you would not test 4 vs. 3 and 3 vs. 2, but still test 4 vs. 1 and 3 vs. 1 (4 vs. 3 and 3 vs. 2 are enclosed by 4 vs. 2: 4 3 2 1). Note that not testing the enclosed means is a procedural rule, and a result of Do Not Test should be treated as if there is no significant difference between the means, even though one may appear to exist.

One Way Analysis of Variance

Dependent Variable: PP2C 62

Equal Variance Test (Brown-Forsythe): Passed (P = 0,140)

**Group Name**	**N**	**Missing**	**Mean**	**Std Dev**	**SEM**
0 ml l^{-1} 24 h	9	0	1,044	0,565	0,188
0 ml l^{-1} 48 h	9	0	1,486	0,632	0,211
4 ml l^{-1} 24 h	9	0	1,861	0,921	0,307
4 ml l^{-1} 48 h	9	0	2,554	0,884	0,295
2 ml l^{-1} 24 h	9	0	0,901	0,308	0,103
2 ml l^{-1} 48 h	9	0	0,627	0,226	0,0752
1 ml l^{-1} 24 h	9	0	1,870	0,995	0,332
1 ml l^{-1} 48 h	9	0	1,811	0,874	0,291

**Source of Variation**	**DF**	**SS**	**MS**	**F**	**P**
Between Groups	7	25,219	3,603	6,784	<0,001
Residual	64	33,988	0,531		
Total	71	59,207			

The differences in the mean values among the treatment groups are greater than would be expected by chance; there is a statistically significant difference (P = <0,001).

Power of performed test with alpha = 0,050: 0,998

All Pairwise Multiple Comparison Procedures (Duncan's Method):

Comparisons for factor: **Treatment**

**Comparison**	**Diff of Means**	**p**	**q**	**P**	**P<0,050**
4 ml l^{-1} 48 vs. 2 ml l^{-1} 48	1,928	8	7,936	<0,001	Yes
4 ml l^{-1} 48 vs. 2 ml l^{-1} 24	1,653	7	6,806	<0,001	Yes
4 ml l^{-1} 48 vs. 0 ml l^{-1} 24	1,510	6	6,216	<0,001	Yes
4 ml l^{-1} 48 vs. 0 ml l^{-1} 48	1,069	5	4,400	0,006	Yes
4 ml l^{-1} 48 vs. 1 ml l^{-1} 48	0,743	4	3,060	0,051	No
4 ml l^{-1} 48 vs. 4 ml l^{-1} 24	0,693	3	2,854	0,060	Do Not Test
4 ml l^{-1} 48 vs. 1 ml l^{-1} 24	0,684	2	2,818	0,051	Do Not Test
1 ml l^{-1} 24 vs. 2 ml l^{-1} 48	1,243	7	5,118	0,002	Yes
1 ml l^{-1} 24 vs. 2 ml l^{-1} 24	0,969	6	3,989	0,014	Yes
1 ml l^{-1} 24 vs. 0 ml l^{-1} 24	0,826	5	3,399	0,034	Yes
1 ml l^{-1} 24 vs. 0 ml l^{-1} 48	0,384	4	1,583	0,315	No
1 ml l^{-1} 24 vs. 1 ml l^{-1} 48	0,0589	3	0,242	0,874	Do Not Test

1 ml l^{-1} 24 vs. 4 ml l^{-1} 24	0,00889	2	0,0366	0,980	Do Not Test
4 ml l^{-1} 24 vs. 2 ml l^{-1} 48	1,234	6	5,082	0,002	Yes
4 ml l^{-1} 24 vs. 2 ml l^{-1} 24	0,960	5	3,952	0,013	Yes
4 ml l^{-1} 24 vs. 0 ml l^{-1} 24	0,817	4	3,362	0,032	Yes
4 ml l^{-1} 24 vs. 0 ml l^{-1} 48	0,376	3	1,546	0,308	Do Not Test
4 ml l^{-1} 24 vs. 1 ml l^{-1} 48	0,0500	2	0,206	0,885	Do Not Test
1 ml l^{-1} 48 vs. 2 ml l^{-1} 48	1,184	5	4,876	0,002	Yes
1 ml l^{-1} 48 vs. 2 ml l^{-1} 24	0,910	4	3,746	0,017	Yes
1 ml l^{-1} 48 vs. 0 ml l^{-1} 24	0,767	3	3,156	0,037	Yes
1 ml l^{-1} 48 vs. 0 ml l^{-1} 48	0,326	2	1,340	0,347	Do Not Test
0 ml l^{-1} 48 vs. 2 ml l^{-1} 48	0,859	4	3,536	0,024	Yes
0 ml l^{-1} 48 vs. 2 ml l^{-1} 24	0,584	3	2,406	0,113	No
0 ml l^{-1} 48 vs. 0 ml l^{-1} 24	0,441	2	1,816	0,204	Do Not Test
0 ml l^{-1} 24 vs. 2 ml l^{-1} 48	0,418	3	1,720	0,257	No
0 ml l^{-1} 24 vs. 2 ml l^{-1} 24	0,143	2	0,590	0,678	Do Not Test
2 ml l^{-1} 24 vs. 2 ml l^{-1} 48	0,274	2	1,130	0,427	Do Not Test

A result of "Do Not Test" occurs for a comparison when no significant difference is found between two means that enclose that comparison. For example, if you had four means sorted in order, and found no difference between means 4 vs. 2, then you would not test 4 vs. 3 and 3 vs. 2, but still test 4 vs. 1 and 3 vs. 1 (4 vs. 3 and 3 vs. 2 are enclosed by 4 vs. 2: 4 3 2 1). Note that not testing the enclosed means is a procedural rule, and a result of Do Not Test should be treated as if there is no significant difference between the means, even though one may appear to exist.

One Way Analysis of Variance

Dependent Variable: MYB23

Equal Variance Test (Brown-Forsythe): Passed (P = 0,182)

**Group Name**	**N**	**Missing**	**Mean**	**Std Dev**	**SEM**
0 ml l^{-1} 24 h	9	0	0,588	0,247	0,0824
0 ml l^{-1} 48 h	9	0	0,964	0,633	0,211
4 ml l^{-1} 24 h	9	0	0,934	0,186	0,0620
4 ml l^{-1} 48 h	9	0	1,744	0,914	0,305
2 ml l^{-1} 24 h	9	0	2,394	0,671	0,224
2 ml l^{-1} 48 h	9	0	1,796	0,821	0,274
1 ml l^{-1} 24 h	9	0	1,277	0,718	0,239
1 ml l^{-1} 48 h	9	0	1,127	0,938	0,313

**Source of Variation**	**DF**	**SS**	**MS**	**F**	**P**
Between Groups	7	21,622	3,089	6,418	<0,001
Residual	64	30,804	0,481		
Total	71	52,426			

The differences in the mean values among the treatment groups are greater than would be expected by chance; there is a statistically significant difference (P = <0,001).

Power of performed test with alpha = 0,050: 0,997

All Pairwise Multiple Comparison Procedures (Duncan's Method) :

Comparisons for factor: **Treatment**

**Comparison**	**Diff of Means**	**p**	**q**	**P**	**P<0,050**
2 ml l^{-1} 24 vs. 0 ml l^{-1} 24	1,807	8	7,812	<0,001	Yes
2 ml l^{-1} 24 vs. 4 ml l^{-1} 24	1,460	7	6,313	<0,001	Yes
2 ml l^{-1} 24 vs. 0 ml l^{-1} 48	1,430	6	6,184	<0,001	Yes
2 ml l^{-1} 24 vs. 1 ml l^{-1} 48	1,268	5	5,482	<0,001	Yes
2 ml l^{-1} 24 vs. 1 ml l^{-1} 24	1,118	4	4,834	0,002	Yes
2 ml l^{-1} 24 vs. 4 ml l^{-1} 48	0,650	3	2,811	0,064	No
2 ml l^{-1} 24 vs. 2 ml l^{-1} 48	0,599	2	2,590	0,072	Do Not Test
2 ml l^{-1} 48 vs. 0 ml l^{-1} 24	1,208	7	5,223	0,001	Yes
2 ml l^{-1} 48 vs. 4 ml l^{-1} 24	0,861	6	3,724	0,022	Yes
2 ml l^{-1} 48 vs. 0 ml l^{-1} 48	0,831	5	3,594	0,024	Yes
2 ml l^{-1} 48 vs. 1 ml l^{-1} 48	0,669	4	2,892	0,065	No
2 ml l^{-1} 48 vs. 1 ml l^{-1} 24	0,519	3	2,244	0,139	Do Not Test

2 ml l^{-1} 48	vs. 4 ml l^{-1} 48	0,0511	2	0,221	0,876	Do Not Test
4 ml l^{-1} 48	vs. 0 ml l^{-1} 24	1,157	6	5,002	0,002	Yes
4 ml l^{-1} 48	vs. 4 ml l^{-1} 24	0,810	5	3,503	0,028	Yes
4 ml l^{-1} 48	vs. 0 ml l^{-1} 48	0,780	4	3,373	0,031	Yes
4 ml l^{-1} 48	vs. 1 ml l^{-1} 48	0,618	3	2,671	0,078	Do Not Test
4 ml l^{-1} 48	vs. 1 ml l^{-1} 24	0,468	2	2,023	0,158	Do Not Test
1 ml l^{-1} 24	vs. 0 ml l^{-1} 24	0,689	5	2,979	0,063	No
1 ml l^{-1} 24	vs. 4 ml l^{-1} 24	0,342	4	1,480	0,348	Do Not Test
1 ml l^{-1} 24	vs. 0 ml l^{-1} 48	0,312	3	1,350	0,374	Do Not Test
1 ml l^{-1} 24	vs. 1 ml l^{-1} 48	0,150	2	0,649	0,648	Do Not Test
1 ml l^{-1} 48	vs. 0 ml l^{-1} 24	0,539	4	2,330	0,138	Do Not Test
1 ml l^{-1} 48	vs. 4 ml l^{-1} 24	0,192	3	0,831	0,584	Do Not Test
1 ml l^{-1} 48	vs. 0 ml l^{-1} 48	0,162	2	0,701	0,622	Do Not Test
0 ml l^{-1} 48	vs. 0 ml l^{-1} 24	0,377	3	1,629	0,283	Do Not Test
0 ml l^{-1} 48	vs. 4 ml l^{-1} 24	0,0300	2	0,130	0,927	Do Not Test
4 ml l^{-1} 24	vs. 0 ml l^{-1} 24	0,347	2	1,499	0,293	Do Not Test

A result of "Do Not Test" occurs for a comparison when no significant difference is found between two means that enclose that comparison. For example, if you had four means sorted in order, and found no difference between means 4 vs. 2, then you would not test 4 vs. 3 and 3 vs. 2, but still test 4 vs. 1 and 3 vs. 1 (4 vs. 3 and 3 vs. 2 are enclosed by 4 vs. 2: 4 3 2 1). Note that not testing the enclosed means is a procedural rule, and a result of Do Not Test should be treated as if there is no significant difference between the means, even though one may appear to exist.

One Way Analysis of Variance

Dependent Variable: ARF19

Equal Variance Test (Brown-Forsythe): Failed (P < 0,050)

**Group Name**	**N**	**Missing**	**Mean**	**Std Dev**	**SEM**
0 ml l^{-1} 24 h	9	0	1,558	0,712	0,237
0 ml l^{-1} 48 h	9	0	1,842	0,967	0,322
4 ml l^{-1} 24 h	9	0	1,962	0,371	0,124
4 ml l^{-1} 48 h	9	0	2,516	0,366	0,122
2 ml l^{-1} 24 h	9	0	1,624	0,768	0,256
2 ml l^{-1} 48 h	9	0	1,439	0,445	0,148
1 ml l^{-1} 24 h	9	0	1,792	0,255	0,0849
1 ml l^{-1} 48 h	9	0	2,288	0,439	0,146

**Source of Variation**	**DF**	**SS**	**MS**	**F**	**P**
Between Groups	7	8,548	1,221	3,541	0,003
Residual	64	22,070	0,345		
Total	71	30,618			

The differences in the mean values among the treatment groups are greater than would be expected by chance; there is a statistically significant difference (P = 0,003).

Power of performed test with alpha = 0,050: 0,848

All Pairwise Multiple Comparison Procedures (Duncan's Method) :

Comparisons for factor: **Treatment**

**Comparison**	**Diff of Means**	**p**	**q**	**P**	**P<0,050**
4 ml l^{-1} 48 vs. 2 ml l^{-1} 48	1,077	8	5,500	<0,001	Yes
4 ml l^{-1} 48 vs. 0 ml l^{-1} 24	0,958	7	4,893	0,003	Yes
4 ml l^{-1} 48 vs. 2 ml l^{-1} 24	0,891	6	4,552	0,005	Yes
4 ml l^{-1} 48 vs. 1 ml l^{-1} 24	0,723	5	3,695	0,021	Yes
4 ml l^{-1} 48 vs. 0 ml l^{-1} 48	0,673	4	3,440	0,028	Yes
4 ml l^{-1} 48 vs. 4 ml l^{-1} 24	0,553	3	2,827	0,062	No
4 ml l^{-1} 48 vs. 1 ml l^{-1} 48	0,228	2	1,164	0,414	Do Not Test
1 ml l^{-1} 48 vs. 2 ml l^{-1} 48	0,849	7	4,337	0,008	Yes
1 ml l^{-1} 48 vs. 0 ml l^{-1} 24	0,730	6	3,729	0,022	Yes
1 ml l^{-1} 48 vs. 2 ml l^{-1} 24	0,663	5	3,389	0,034	Yes
1 ml l^{-1} 48 vs. 1 ml l^{-1} 24	0,496	4	2,532	0,107	No
1 ml l^{-1} 48 vs. 0 ml l^{-1} 48	0,446	3	2,276	0,133	Do Not Test

1 ml l^{-1} 48 vs. 4 ml l^{-1} 24	0,326	2	1,663	0,244	Do Not Test
4 ml l^{-1} 24 vs. 2 ml l^{-1} 48	0,523	6	2,674	0,102	No
4 ml l^{-1} 24 vs. 0 ml l^{-1} 24	0,404	5	2,066	0,200	Do Not Test
4 ml l^{-1} 24 vs. 2 ml l^{-1} 24	0,338	4	1,726	0,273	Do Not Test
4 ml l^{-1} 24 vs. 1 ml l^{-1} 24	0,170	3	0,868	0,568	Do Not Test
4 ml l^{-1} 24 vs. 0 ml l^{-1} 48	0,120	2	0,613	0,666	Do Not Test
0 ml l^{-1} 48 vs. 2 ml l^{-1} 48	0,403	5	2,061	0,202	Do Not Test
0 ml l^{-1} 48 vs. 0 ml l^{-1} 24	0,284	4	1,453	0,357	Do Not Test
0 ml l^{-1} 48 vs. 2 ml l^{-1} 24	0,218	3	1,113	0,464	Do Not Test
0 ml l^{-1} 48 vs. 1 ml l^{-1} 24	0,0500	2	0,255	0,857	Do Not Test
1 ml l^{-1} 24 vs. 2 ml l^{-1} 48	0,353	4	1,805	0,252	Do Not Test
1 ml l^{-1} 24 vs. 0 ml l^{-1} 24	0,234	3	1,198	0,430	Do Not Test
1 ml l^{-1} 24 vs. 2 ml l^{-1} 24	0,168	2	0,857	0,547	Do Not Test
2 ml l^{-1} 24 vs. 2 ml l^{-1} 48	0,186	3	0,948	0,533	Do Not Test
2 ml l^{-1} 24 vs. 0 ml l^{-1} 24	0,0667	2	0,341	0,811	Do Not Test
0 ml l^{-1} 24 vs. 2 ml l^{-1} 48	0,119	2	0,607	0,669	Do Not Test

A result of "Do Not Test" occurs for a comparison when no significant difference is found between two means that enclose that comparison. For example, if you had four means sorted in order, and found no difference between means 4 vs. 2, then you would not test 4 vs. 3 and 3 vs. 2, but still test 4 vs. 1 and 3 vs. 1 (4 vs. 3 and 3 vs. 2 are enclosed by 4 vs. 2: 4 3 2 1). Note that not testing the enclosed means is a procedural rule, and a result of Do Not Test should be treated as if there is no significant difference between the means, even though one may appear to exist.

One Way Analysis of Variance

Dependent Variable: NH30

Equal Variance Test (Brown-Forsythe): Passed (P = 0,464)

Group Name	N	Missing	Mean	Std Dev	SEM
0 ml l^{-1} 24 h	9	0	1,303	0,608	0,203
0 ml l^{-1} 48 h	9	3	2,503	0,232	0,0948
4 ml l^{-1} 24 h	9	0	1,741	0,324	0,108
4 ml l^{-1} 48 h	9	2	2,036	0,459	0,173
2 ml l^{-1} 24 h	9	0	2,272	0,325	0,108
2 ml l^{-1} 48 h	9	2	2,344	0,528	0,199
1 ml l^{-1} 24 h	9	0	2,151	0,239	0,0798
1 ml l^{-1} 48 h	9	3	2,732	0,393	0,160

Source of Variation	DF	SS	MS	F	P
Between Groups	7	10,965	1,566	9,325	<0,001
Residual	54	9,071	0,168		
Total	61	20,036			

The differences in the mean values among the treatment groups are greater than would be expected by chance; there is a statistically significant difference (P = <0,001).

Power of performed test with alpha = 0,050: 1,000

All Pairwise Multiple Comparison Procedures (Duncan's Method) :

Comparisons for factor: **Treatment**

Comparison	Diff of Means	p	q	P	P<0,050
1 ml l^{-1} 48 vs. 0 ml l^{-1} 24	1,428	8	9,351	<0,001	Yes
1 ml l^{-1} 48 vs. 4 ml l^{-1} 24	0,991	7	6,485	<0,001	Yes
1 ml l^{-1} 48 vs. 4 ml l^{-1} 48	0,696	6	4,316	0,008	Yes
1 ml l^{-1} 48 vs. 1 ml l^{-1} 24	0,581	5	3,801	0,018	Yes
1 ml l^{-1} 48 vs. 2 ml l^{-1} 24	0,459	4	3,008	0,056	No
1 ml l^{-1} 48 vs. 2 ml l^{-1} 48	0,387	3	2,403	0,114	Do Not Test
1 ml l^{-1} 48 vs. 0 ml l^{-1} 48	0,228	2	1,365	0,339	Do Not Test
0 ml l^{-1} 48 vs. 0 ml l^{-1} 24	1,200	7	7,856	<0,001	Yes
0 ml l^{-1} 48 vs. 4 ml l^{-1} 24	0,762	6	4,990	0,002	Yes
0 ml l^{-1} 48 vs. 4 ml l^{-1} 48	0,468	5	2,900	0,071	No
0 ml l^{-1} 48 vs. 1 ml l^{-1} 24	0,352	4	2,306	0,143	Do Not Test
0 ml l^{-1} 48 vs. 2 ml l^{-1} 24	0,231	3	1,513	0,319	Do Not Test

0 ml l^{-1} 48 vs. 2 ml l^{-1} 48	0,159	2	0,986	0,489	Do Not Test
2 ml l^{-1} 48 vs. 0 ml l^{-1} 24	1,041	6	7,127	<0,001	Yes
2 ml l^{-1} 48 vs. 4 ml l^{-1} 24	0,603	5	4,130	0,010	Yes
2 ml l^{-1} 48 vs. 4 ml l^{-1} 48	0,309	4	1,992	0,206	Do Not Test
2 ml l^{-1} 48 vs. 1 ml l^{-1} 24	0,193	3	1,323	0,384	Do Not Test
2 ml l^{-1} 48 vs. 2 ml l^{-1} 24	0,0721	2	0,493	0,729	Do Not Test
2 ml l^{-1} 24 vs. 0 ml l^{-1} 24	0,969	5	7,092	<0,001	Yes
2 ml l^{-1} 24 vs. 4 ml l^{-1} 24	0,531	4	3,888	0,013	Yes
2 ml l^{-1} 24 vs. 4 ml l^{-1} 48	0,237	3	1,619	0,287	Do Not Test
2 ml l^{-1} 24 vs. 1 ml l^{-1} 24	0,121	2	0,886	0,534	Do Not Test
1 ml l^{-1} 24 vs. 0 ml l^{-1} 24	0,848	4	6,205	<0,001	Yes
1 ml l^{-1} 24 vs. 4 ml l^{-1} 24	0,410	3	3,001	0,049	Yes
1 ml l^{-1} 24 vs. 4 ml l^{-1} 48	0,115	2	0,790	0,579	Do Not Test
4 ml l^{-1} 48 vs. 0 ml l^{-1} 24	0,732	3	5,015	0,001	Yes
4 ml l^{-1} 48 vs. 4 ml l^{-1} 24	0,295	2	2,017	0,160	No
4 ml l^{-1} 24 vs. 0 ml l^{-1} 24	0,438	2	3,204	0,028	Yes

A result of "Do Not Test" occurs for a comparison when no significant difference is found between two means that enclose that comparison. For example, if you had four means sorted in order, and found no difference between means 4 vs. 2, then you would not test 4 vs. 3 and 3 vs. 2, but still test 4 vs. 1 and 3 vs. 1 (4 vs. 3 and 3 vs. 2 are enclosed by 4 vs. 2: 4 3 2 1). Note that not testing the enclosed means is a procedural rule, and a result of Do Not Test should be treated as if there is no significant difference between the means, even though one may appear to exist.

One Way Analysis of Variance

Dependent Variable: YUCCA6

Equal Variance Test (Brown-Forsythe): Passed (P = 0,829)

Group Name	N	Missing	Mean	Std Dev	SEM
0 ml l^{-1} 24 h	9	0	1,189	0,519	0,173
0 ml l^{-1} 48 h	9	0	1,258	0,562	0,187
4 ml l^{-1} 24 h	9	0	1,503	0,607	0,202
4 ml l^{-1} 48 h	9	0	2,087	0,682	0,227
2 ml l^{-1} 24 h	9	0	2,126	0,567	0,189
2 ml l^{-1} 48 h	9	0	1,688	0,352	0,117
1 ml l^{-1} 24 h	9	3	2,937	0,541	0,221
1 ml l^{-1} 48 h	9	0	1,977	0,548	0,183

Source of Variation	DF	SS	MS	F	P
Between Groups	7	16,639	2,377	7,723	<0,001
Residual	61	18,775	0,308		
Total	68	35,414			

The differences in the mean values among the treatment groups are greater than would be expected by chance; there is a statistically significant difference (P = <0,001).

Power of performed test with alpha = 0,050: 1,000

All Pairwise Multiple Comparison Procedures (Duncan's Method) :

Comparisons for factor: **Treatment**

Comparison	Diff of Means	p	q	P	P<0,050
1 ml l^{-1} 24 vs. 0 ml l^{-1} 24	1,748	8	8,453	<0,001	Yes
1 ml l^{-1} 24 vs. 0 ml l^{-1} 48	1,679	7	8,120	<0,001	Yes
1 ml l^{-1} 24 vs. 4 ml l^{-1} 24	1,433	6	6,932	<0,001	Yes
1 ml l^{-1} 24 vs. 2 ml l^{-1} 48	1,249	5	6,040	<0,001	Yes
1 ml l^{-1} 24 vs. 1 ml l^{-1} 48	0,960	4	4,643	0,003	Yes
1 ml l^{-1} 24 vs. 4 ml l^{-1} 48	0,850	3	4,111	0,007	Yes
1 ml l^{-1} 24 vs. 2 ml l^{-1} 24	0,811	2	3,923	0,007	Yes
2 ml l^{-1} 24 vs. 0 ml l^{-1} 24	0,937	7	5,065	0,002	Yes
2 ml l^{-1} 24 vs. 0 ml l^{-1} 48	0,868	6	4,693	0,004	Yes
2 ml l^{-1} 24 vs. 4 ml l^{-1} 24	0,622	5	3,365	0,036	Yes
2 ml l^{-1} 24 vs. 2 ml l^{-1} 48	0,438	4	2,367	0,132	No
2 ml l^{-1} 24 vs. 1 ml l^{-1} 48	0,149	3	0,805	0,596	Do Not Test

$2\ ml\ l^{-1}$ 24 vs. $4\ ml\ l^{-1}$ 48	0,0389	2	0,210	0,882	Do Not Test
$4\ ml\ l^{-1}$ 48 vs. $0\ ml\ l^{-1}$ 24	0,898	6	4,855	0,003	Yes
$4\ ml\ l^{-1}$ 48 vs. $0\ ml\ l^{-1}$ 48	0,829	5	4,482	0,005	Yes
$4\ ml\ l^{-1}$ 48 vs. $4\ ml\ l^{-1}$ 24	0,583	4	3,154	0,044	Yes
$4\ ml\ l^{-1}$ 48 vs. $2\ ml\ l^{-1}$ 48	0,399	3	2,157	0,155	Do Not Test
$4\ ml\ l^{-1}$ 48 vs. $1\ ml\ l^{-1}$ 48	0,110	2	0,595	0,676	Do Not Test
$1\ ml\ l^{-1}$ 48 vs. $0\ ml\ l^{-1}$ 24	0,788	5	4,260	0,008	Yes
$1\ ml\ l^{-1}$ 48 vs. $0\ ml\ l^{-1}$ 48	0,719	4	3,887	0,013	Yes
$1\ ml\ l^{-1}$ 48 vs. $4\ ml\ l^{-1}$ 24	0,473	3	2,560	0,092	No
$1\ ml\ l^{-1}$ 48 vs. $2\ ml\ l^{-1}$ 48	0,289	2	1,562	0,274	Do Not Test
$2\ ml\ l^{-1}$ 48 vs. $0\ ml\ l^{-1}$ 24	0,499	4	2,698	0,086	No
$2\ ml\ l^{-1}$ 48 vs. $0\ ml\ l^{-1}$ 48	0,430	3	2,325	0,126	Do Not Test
$2\ ml\ l^{-1}$ 48 vs. $4\ ml\ l^{-1}$ 24	0,184	2	0,997	0,483	Do Not Test
$4\ ml\ l^{-1}$ 24 vs. $0\ ml\ l^{-1}$ 24	0,314	3	1,700	0,263	Do Not Test
$4\ ml\ l^{-1}$ 24 vs. $0\ ml\ l^{-1}$ 48	0,246	2	1,328	0,352	Do Not Test
$0\ ml\ l^{-1}$ 48 vs. $0\ ml\ l^{-1}$ 24	0,0689	2	0,373	0,793	Do Not Test

A result of "Do Not Test" occurs for a comparison when no significant difference is found between two means that enclose that comparison. For example, if you had four means sorted in order, and found no difference between means 4 vs. 2, then you would not test 4 vs. 3 and 3 vs. 2, but still test 4 vs. 1 and 3 vs. 1 (4 vs. 3 and 3 vs. 2 are enclosed by 4 vs. 2: 4 3 2 1). Note that not testing the enclosed means is a procedural rule, and a result of Do Not Test should be treated as if there is no significant difference between the means, even though one may appear to exist.
